# Response to Hypomethylating Agents in Myelodysplastic Syndrome Is Associated With Emergence of Novel TCR Clonotypes

**DOI:** 10.3389/fimmu.2021.659625

**Published:** 2021-04-12

**Authors:** Hussein A. Abbas, Patrick K. Reville, Xianli Jiang, Hui Yang, Alexandre Reuben, Jin Seon Im, Latasha Little, Jefferson C. Sinson, Ken Chen, Andrew Futreal, Guillermo Garcia-Manero

**Affiliations:** ^1^ Division of Cancer Medicine, Medical Oncology Fellowship, University of Texas MD Anderson Cancer Center, Houston, TX, United States; ^2^ Department of Bioinformatics and Computational Biology, University of Texas MD Anderson Cancer Center, Houston, TX, United States; ^3^ Department of Leukemia, University of Texas MD Anderson Cancer Center, Houston, TX, United States; ^4^ Department of Thoracic/Head & Neck Medical Oncology, University of Texas MD Anderson Cancer Center, Houston, TX, United States; ^5^ Department of Stem Cell Transplantation and Cellular Therapy, University of Texas MD Anderson Cancer Center, Houston, TX, United States; ^6^ Graduate School of Biomedical Sciences, The University of Texas MD Anderson Cancer Center, Houston, TX, United States; ^7^ Department of Genomic Medicine, The University of Texas MD Anderson Cancer Center, Houston, TX, United States

**Keywords:** myelodysplastic syndrome, T-cell repertoire, diversity, clonality, antigen recognition

## Abstract

Aberrant T-cell function is implicated in the pathogenesis of myelodysplastic syndrome (MDS). Monitoring the T-cell receptor (TCR) repertoire can provide insights into T-cell adaptive immunity. Previous studies found skewed TCR repertoires in MDS compared to healthy patients; however these studies that leverage mRNA-based spectratyping have limitations. Furthermore, evaluating the TCR repertoire in context of hypomethylating agents (HMAs) treatment can provide insights into the dynamics of T-cell mediated responses in MDS. We conducted immunosequencing of the CDR3 regions of TCRβ chains in bone marrows of 11 MDS patients prior to treatment (n=11 bone marrows prior to treatment), and in at least 2 timepoints for each patient following treatment (n=26 bone marrow aspirates post-treatment) with (HMA), alongside analyzing bone marrows from 4 healthy donors as controls. TCR repertoires in MDS patients were more clonal and less diverse than healthy donors. However, unlike previous reports, we did not observe significant skewness in CDR3 length or spectratyping. The global metrics of TCR profiling including richness, clonality, overlaps were not significantly changed in responders or non-responders following treatment with HMAs. However, we found an emergence of novel clonotypes in MDS patients who responded to treatment, while non-responders had a higher frequency of contracted clonotypes following treatment. By applying GLIPH2 for antigen prediction, we found rare TCR specificity clusters shared by TCR clonotypes from different patients at pre- or following treatment. Our data show clear differences in TCR repertoires of MDS compared with healthy patients and that novel TCR clonotype emergence in response to HMA therapy was correlated with response. This suggests that response to HMA therapy may be partially driven by T-cell mediated immunity and that the immune-based therapies, which target the adaptive immune system, may play a significant role in select patients with MDS.

## Introduction

Myelodysplastic syndrome (MDS) is a clonal hematologic disorder characterized by dysplastic hematopoiesis and increased risk of transformation into acute myeloid leukemia (AML) (1). Hypomethylating agents (HMA), such as azacitidine and decitabine, are the mainstay of MDS treatment leading to decreased transfusion requirements and improved quality of life (1, 2). Several studies have implicated T-cell dysfunction with MDS, consistent with the finding that several MDS patients respond to immunosuppressive treatments (3–5). For instance, T-cell dysregulation (6, 7), T-cell inhibition of hematopoietic precursors (8), and auto-immune T-cell activity may lead to selection of dysplastic clones in MDS (6). Moreover, 10% of MDS patients have autoimmune clinical manifestations including vasculitis, dermatitis and nephritis (9). Evaluating T-cell dynamics in response to HMA treatment in MDS is warranted to evaluate whether clinical responses are mediated *via* T-cell modulation.

T-cell receptor (TCR) repertoire analysis serves as a surrogate to investigate the immune-based responses to treatment and offers a snapshot of T-cell diversity and versatility (10–12). TCRs are highly polymorphic surface receptors that recognize antigenic peptides presented in major histocompatibility complex (MHC) (13). TCR diversity is generated through somatic recombination of variable (V), diversity (D), and joining (J) gene segments along with pseudorandom insertions and deletions of nucleic acids at the joining regions (14). This process has the potential to create over 10^15^ TCR clonotypes with estimates of unique T cells in a humans ranging from 10^6^ - 10^11^ (15). By deep sequencing of TCR repertoires, one can obtain a view into the antigenic exposure of an individual (16). Expansions and contractions of T cell clonotypes can be tracked using this method whereby activated and expanding T cells against specific cognate antigens lead to skewed TCR repertoires.

Previous studies have reported a skewed TCR repertoire in MDS patients. Using spectratyping which leverages complementary determining region 3 (CDR3) length (17–19), the skewness of TCR repertoire in MDS improved following treatment with azacitidine (20). However, while the length of the *CDR3* distribution is one parameter of T-cell diversity, most of the variability lies in the CDR3 sequence that is encoded within the V(D)J region, represented by *β* sequences, which comes in contact with an antigenic peptide (21, 22). Further, previous studies leveraged mRNA-based complimentary DNA sequencing which is biased due to different mRNA content per cell and is affected by RNA input (23). Therefore, deep sequencing of the genomic *CDR3 beta-*region would allow a more accurate representation of the TCR diversity (23).

To investigate T-cell dynamics in the setting of HMA therapy, we applied TCR *β*-region sequencing in 11 MDS patients before (n=11 bone marrows) and after (n=26 bone marrows) treatment with HMA. We also leveraged a public dataset to compare the MDS TCR diversity to healthy donors (n=4), and applied GLIPH2 pipeline (24, 25) to evaluate homologous clonotypes that could recognize the same antigen.

## Materials and Methods

### Patient Population and Outcomes

A total of 11 patients with MDS who received azacitidine or decitabine and had bone marrow samples before and after treatment with hypomethylating agents on protocols at University of Texas M D Anderson Cancer Center were included in this study. TCR immunosequencing data from 4 healthy controls was downloaded from Adaptive website (ref) and were included as controls. MDS patients with complete response (CR) attained <5% myeloblasts with normal maturation of all cell lines with recovery of peripheral counts (hemoglobin >11 g/dL; ANC>1000/mm3 without myeloid growth factor support; platelets >=100,000/mm3 without thrombopoietic support). Hematologic improvement (HI) response was based on increase in hemoglobin of >=1.5 g/dL without transfusions. Patients with no response did not meet the criteria for CR or HI. CR/HI were considered as responders, while no response patients were considered as non-responders. All patients were treated on an IRB-approved protocol and consented for the research study. Additionally, 4 healthy bone marrow sample (age 18-35 years) TCR data was downloaded from immuneACCESS at https://clients.adaptivebiotech.com/pub/bone-marrow-healthy-adults-control.

### Library Preparation for TCR Immunosequencing

Genomic DNA was extracted from whole bone marrows. Quality assessment of samples was done with Agilent TapeStation genomic DNA screentapes, Thermo Scientific NanoDrop OneC and Quant-iT™ PicoGreen™ dsDNA Assay kit. Adaptive’s hsTCRB kit was used to detect rearranged TCRB gene sequences in the genomic DNA. Libraries were made using multiplex PCR primers, which targeted the complementary determining region 3 (CDR3) of human TCRβ gene following rearrangement of the variable (V), diversity (D) and joining (J) gene segments.

### First PCR

A survey resolution was done and used 2 replicates per sample during the first step PCR and a total of 400 ng of DNA or 200 ng per replicate for the first PCR set up. Using QIAGEN Multiplex PCR Kit, 31 cycles of the first PCR amplified highly variable CDR3 region, using V- and J-gene specific primers. Universal adapters at the end of the V- and J-gene specific primers served as a target for the addition of unique DNA barcodes in the second PCR. The amplicons were then purified using a bead-based system to remove residual primers and unamplified targets and were run on an agarose gel to determine that the correct products were amplified.

### Second PCR

The barcodes that were selected in the sample manifest and Illumina adapters were added to each PCR replicate during the 8 cycle second PCR. The libraries were then purified using a bead-based system similar to the first PCR to remove residual primers and unamplified targets. Another agarose gel check was performed to determine that the barcodes and Illumina adapters were added during the second PCR. Equal volume of sequencing ready libraries were then pooled and ran on Agilent D1000 screen tapes to determine the size and the size adjusted concentration. The libraries were quantified prior to sequencing using the Applied Biosystems QuantStudio 6 and KAPA Biosystems library quantification kit.

### Miseq Sequencing

Based on the qPCR results, approximately 15 pM of the pooled libraries were loaded onto the Miseq Sequencing System for a single end read which includes a 156 cycle Read 1 and a 15 cycle Index 1 read run.

### Post Sequencing

Raw sequences output from the Miseq was transferred to Adaptive’s immunoSEQ Data Assistant, where the data was processed to report the normalized and annotated TCRB repertoire profile for each sample. The data was then posted to the immunoSEQ Analyzer account for evaluation of immunosequencing data. Data was downloaded and then analyzed using in R statistical software.

### TCR Analysis and Clonotype Comparison

The clonotype landscape was analyzed using the immunArch R package version 0.6.5 (http://doi.org/10.5281/zenodo.3367200) and Adaptive BioAnalyzer. Downsampling was utilized to define repertoire richness, whereby repertoires were downsampled to the number of clones that the smallest repertoire has. For data where there are two groups, the Wilcoxon rank sum test was performed to test for a difference in mean rank values between two groups. In case there are more than two groups, the Kruskal-Wallis test was performed to tests whether samples from different groups originated from the same distribution. Adjustment for multiple comparisons p-values per analysis was done using the Holm-Bonferroni correction method. To compare the number of contracted/expanded/novel clonotypes occurred in responders with that in non-responders, Fisher’s exact test was implemented by using the count of one clonotype before treatment, total count of all clonotypes before treatment, count of clonotype after treatment and total count of clonotypes after treatment. Expanded clonotypes had significantly larger frequency compared to prior treatment, while novel clonotypes were only detected after treatment with frequency of 0 prior to treatment. Contracted clonotypes had significantly lower frequency after treatment. P-values ≤ 0.05 were considered statistically significant. All statistical analyses were performed using R software.

### GLIPH (Grouping of Lymphocyte Interactions by Paratope Hotspots) Analysis

To identify T-cell specificity groups, GLIPH2 (24, 25) was used to cluster CDR3 β-chain sequences. Briefly, we ran the analysis for unique CDR3 sequences from only significantly changed clonotypes and 780 of them meet analysis criteria. Parameters were set as: simulation_depth=1000, kmer_min_depth=3. Clonotypes with missing or short (n<5) beta- CDR3 sequence were excluded from the clustering analysis. The output of GLIPH2 analyses was visualized with the iGraph package in R software. Clonotypes that belong to same cluster were connected by edges and clonotypes that have no shared clones were shown as single nodes.

## Results

### Patient Cohort

We conducted immunosequencing of the CDR3 regions of TCRβ chains of bone marrow genomic DNA from 11 (9/11 male; 2/11 female) MDS patients prior to (n=11) and following (n=26) treatment with the hypomethylating agents azacitidine (8/11) or decitabine (3/11) at different treatment timepoints. Clinical and demographic data is summarized in **Table 1**. Briefly, the average age was 72.2 ± 6.3 years. The most common cytogenetic profile was diploid in 8/11 (73%) patients. The most common mutation was *TET2* in 5/11 (45%) of patients. A total of 6/11 (54%) patients were responders (CR or HI) and 5/11 (46%) patients were non-responders. We also analyzed the immunosequencing of the CDR3 regions of TCRβ from 4 publicly available healthy bone marrows from healthy adult donors (age 18-35 years).

**Table 1:** Clinical and demographic characteristics.

Characteristic	Overall, N = 11	CR/HI, N = 6	No Response, N = 5	p-value_1_	q-value_2_
**Gender, n/N (%)**				>0.9	>0.9
Female	2/11 (18%)	1/6 (17%)	1/5 (20%)		
Male	9/11 (82%)	5/6 (83%)	4/5 (80%)		
**Age, Mean+/-SD**	72.2+/-6.3	70.7+/-6.7	74.0+/-6.0	0.3	>0.9
**Hemoglobin (g/dL), Mean+/-SD**	9.2+/-1.5	9.5+/-1.9	8.8+/-0.5	0.7	>0.9
**Platelets (K/uL), Mean+/-SD**	137.6+/-152.7	134.3+/-151.3	141.6+/-172.1	>0.9	>0.9
**ANC (K.uL), Mean+/-SD**	4.0+/-3.9	4.9+/-5.1	3.0+/-1.9	>0.9	>0.9
**BM Blast (%), Mean+/-SD**	4.3+/-3.3	3.0+/-1.8	5.8+/-4.1	0.2	>0.9
**Cytogenetics, n/N (%)**				0.7	>0.9
8+	2/11 (18%)	1/6 (17%)	1/5 (20%)		
Diploid	8/11 (73%)	5/6 (83%)	3/5 (60%)		
Miscellaneous	1/11 (9.1%)	0/6 (0%)	1/5 (20%)		
***ASXL1*, n/N (%)**	4/11 (36%)	3/6 (50%)	1/5 (20%)	0.5	>0.9
***TET2*, n/N (%)**	5/11 (45%)	3/6 (50%)	2/5 (40%)	>0.9	>0.9
***RUNX1*, n/N (%)**	4/11 (36%)	2/6 (33%)	2/5 (40%)	>0.9	>0.9
**IPSS, n/N (%)**				0.5	>0.9
Low	4/11 (36%)	3/6 (50%)	1/5 (20%)		
INT-1	6/11 (55%)	3/6 (50%)	3/5 (60%)		
High	1/11 (9.1%)	0/6 (0%)	1/5 (20%)		
**HMA, n/N (%)**				>0.9	>0.9
Azacitidine	8/11 (73%)	4/6 (67%)	4/5 (80%)		
Decitabine	3/11 (27%)	2/6 (33%)	1/5 (20%)		

_1_Statistical tests performed: Fisher’s exact test; Wilcoxon rank-sum test.

_2_False discovery rate correction for multiple testing.

ANC, Absolute Neutrophil Count; BM, Bone marrow; IPSS, International Prognostic Scoring System; HMA, Hypomethylating agent.

### Bone Marrows From MDS Patients Are Less Diverse but More Clonal Compared to Healthy Bone Marrows

**Figure 1:**
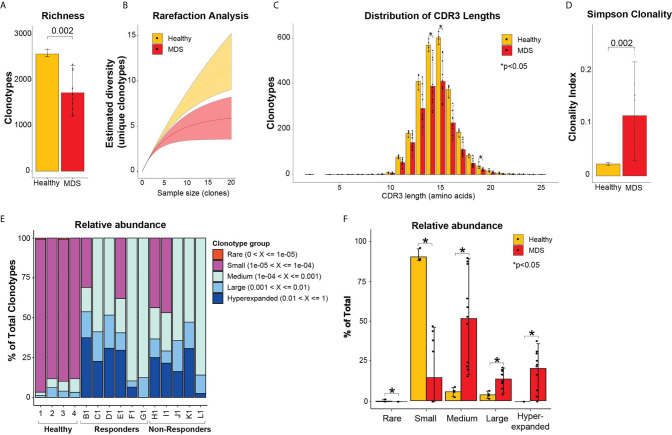
Diversity TCR metrics in healthy and pre-treatment MDS bone marrows. **(A)** Richness and **(B)** rarefaction analysis between healthy and pre-treatment MDS bone marrows for estimation of diversity. **(C)** Distribution of CDR3 lengths in healthy and pre-treatment MDS bone marrows. **(D)** Simpson clonality index as measurement of degree of clonality. **(E)** Relative abundance of the different clonotype groups based on the frequency of clonotype. **(F)** Barplot representation of the relative abundance measurements in **(E)**. *p < 0.05.

To assess the diversity of clonotypes in the bone marrows of MDS patients prior to treatment compared to healthy donor bone marrows, we measured the repertoire richness. Healthy bone marrows had significantly higher number of clonotypes compared to MDS patients (2569 ± 77 vs 1719 ± 397 clonotypes, p = 0.0002) (**Figure 1A**). Further, we performed rarefaction analysis that assesses the repertoire richness *via* extrapolation from sampling results and found more unique clonotypes per clone sample size in the healthy bone marrows compared to MDS (**Figure 1B**). We next evaluated the CDR3 length and found no significant skewing in the amino acid length distributions between healthy and MDS patients (**Figure 1C** **)**. However, more clonotypes were found in healthy donors compared to MDS patients at all CDR3 lengths and significantly more at lengths of 14, 15 and 19 amino acids (**Figure 1C**) consistent with higher richness in healthy bone marrows. These findings indicate that the TCR repertoire of MDS patients is significantly less diverse than healthy donors, although these findings may be hampered by the healthy donors being younger than MDS patients.

To evaluate clonality, we utilized the Simpson clonality index, which is less affected by differences in sample size. We found that MDS patients had significantly higher clonality compared to healthy donors (0.12 ± 0.07 vs 0.02 ± 0.002, respectively, p=0.002) (**Figure 1D**). We next evaluated the relative abundance by measuring the proportion of clonotypes with specific frequencies in the homeostatic space ranging from rare clonotypes (<1x10^-5^) to hyper-expanded (>0.01) (**Figures 1E, F**). The majority of the healthy repertoire was occupied by small clonotype groups, whereas the repertoire of MDS patients was primarily occupied by medium, hyperexpanded then large clonotype groups (p<0.05 for all) (**Figures 1E, F**). These findings support that MDS patients have highly clonal repertoires that are dominated by medium, large, and hyperexpanded clonotypes, while the diversity of their clonotypes is significantly less compared to healthy bone marrows.

### Treatment With HMA Does Not Alter Global Repertoires in MDS

**Figure 2:**
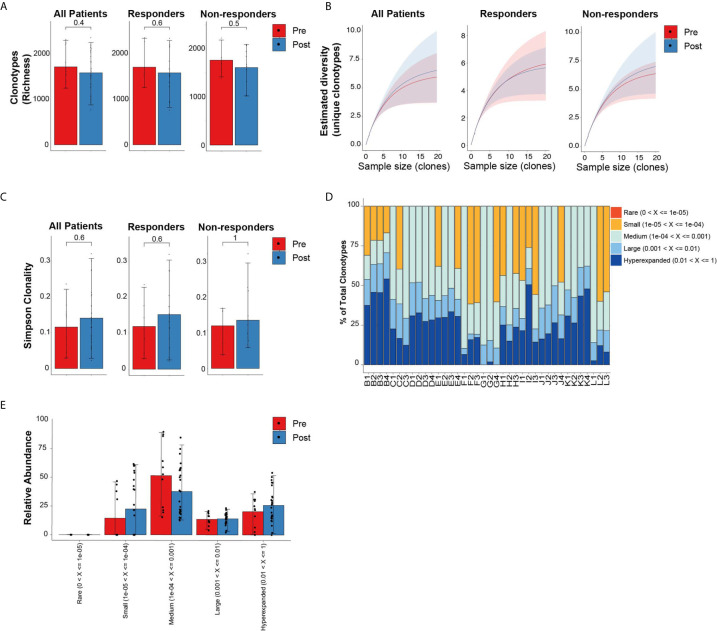
Diversity TCR metrics in MDS patients prior to and following treatment with hypomethylating agents. **(A)** Richness and **(B)** rarefaction analysis in all patients and by response groups. **(C)** Simpson clonality index as measurement of degree of clonality. **(D)** Relative abundance of the different clonotype groups based on the frequency of clonotype. **(E)** Barplot representation of the relative abundance measurements in **(D)**.

Similar to the analysis comparing newly diagnosed MDS to healthy donor bone marrows, we evaluated the global repertoire architecture in MDS prior to and following treatment with HMA (azacitidine or decitabine). All 11 MDS patients (6 responders and 5 non-responders) had baseline bone marrows as well as 2 to 3 bone marrows at different post-treatment timepoints allowing us to assess longitudinal changes in TCR repertoires. Following treatment, there were no significant differences in the diversity metrics of TCR repertoires based on richness (**Figure 2A**) nor rarefaction estimated diversity (**Figure 2B**) across all patients and within response groups. We also did not identify any skewing based on either CDR3 length (**Supplementary Figures 1A–C**) or spectratyping (**Supplementary Figure 2** **)** following treatment. We did not detect differences in overall clonality following treatment and within response groups (**Figure 2C**). The relative abundance of clonotypes at different timepoints was mostly unchanged (**Figures 2D, E** **)**. However, some patients had higher abundance of smaller clonotypes following treatment (patients C, F and L), while others had higher abundance of their large or hyperexpanded clonotypes following treatment (patients H and I).

### Morisita Index Demonstrates Stable TCR Repertoire Profiles Following Treatments

**Figure 3:**
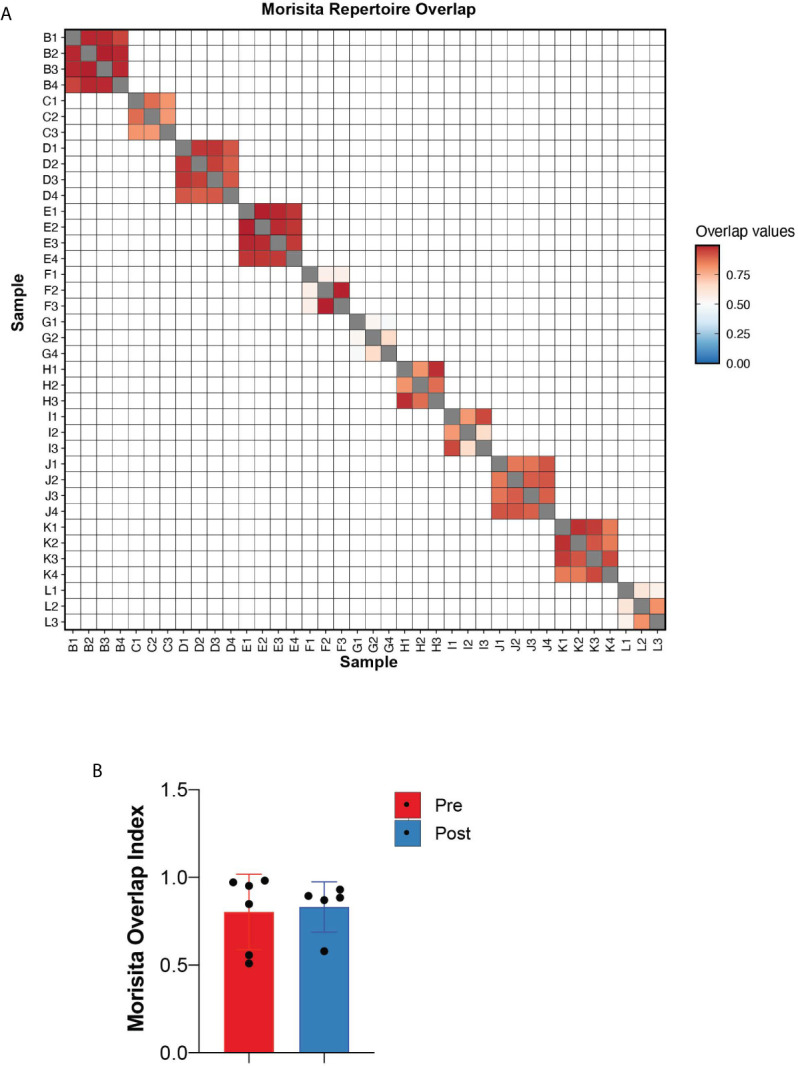
Repertoire overlap. **(A)** Heatmap of the Morisita repertoire overlap across all patient samples. **(B)** Morisita overlap index of MDS bone marrows prior to and following HMA treatment.

To quantitate the similarity and overlap between TCR repertoires at different timepoints, we utilized the Morisita overlap index (ranges from 0 to 1) which measures the similarity (index closer to 1) of TCR clonotypes while accounting for the abundance of T-cell rearrangements (12). Except for patients F and G (both responders with hematologic improvement), the Morisita overlap index was consistently >0.8 suggesting a high degree of TCR similarity between samples of the same patient (**Figure 3A**). Relative to pre-treatment, there were no significant differences in the Morisita overlap index of post-treatment samples between responders and non-responders to HMA treatment (**Figure 3B**). These results suggest that treatment with HMA does not change the global TCR repertoire structure in MDS.

### Assessment of Individual Clonotype Frequencies Reveals Dynamic Changes in Responders and Non-Responders to HMA Treatment

**Figure 4:**
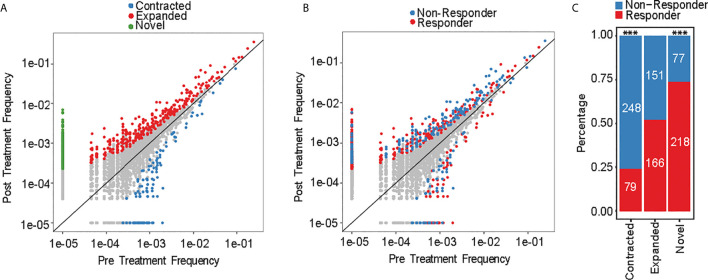
Clonotype frequency assessment. **(A)** Scatterplots of clonotypes change of pre- versus post-treatment and by **(B)** response groups. **(C)** Number of novel, expanded and contracted clonotypes by response group. ***p < 0.001.

Aggregate TCR repertoire analysis can mask individual clonotype dynamics that can drive important and clinically relevant immune activity against MDS. We therefore conducted clonotype tracking across the top 10 clonotypes at pre-treatment (**Supplementary Figures 3A–K** **)** and at any of the treatment timepoints (**Supplementary Figures 4A–K**). There was no specific pattern for changes that occurred by response groups. For instance, some of the responders had expansions of their top 10 clonotypes, while other patients had contraction of their top 10 clonotypes. However, this approach is biased as it evaluates only 10 clonotypes, which constitute <0.1% of all the detected clonotypes. We therefore measured the frequencies of each clonotype at pre-treatment versus post treatment to evaluate the dynamic changes in individual clonotypes. We identified 939 significantly changed clonotypes as novel (295/939; 31%), expanded (317/939; 34%) or contracted (327/939; 35%) (**Figure 4A**). 72% of contracted clonotypes were not detected at post-treatment, while the novel (not detected at pre-treatment) and expanded clonotypes were plotted separately and contained similar number of clones (317 and 295, respectively). When evaluating each clonotype by response group (**Figure 4B**), there was significantly higher abundance of contracted clonotypes in non-responders compared to responders (76%, p-value < 2.2e-16), while significantly more novel clonotypes emerged in responders (74%, p-value = 1.5e-10) (**Figure 4C**). These findings suggest that HMA can induce changes in individual clonotypes and lead to emergence of new clonotypes in responders, while non-responders had a contracted clonotype repertoire following treatment.

**Figure 5:**
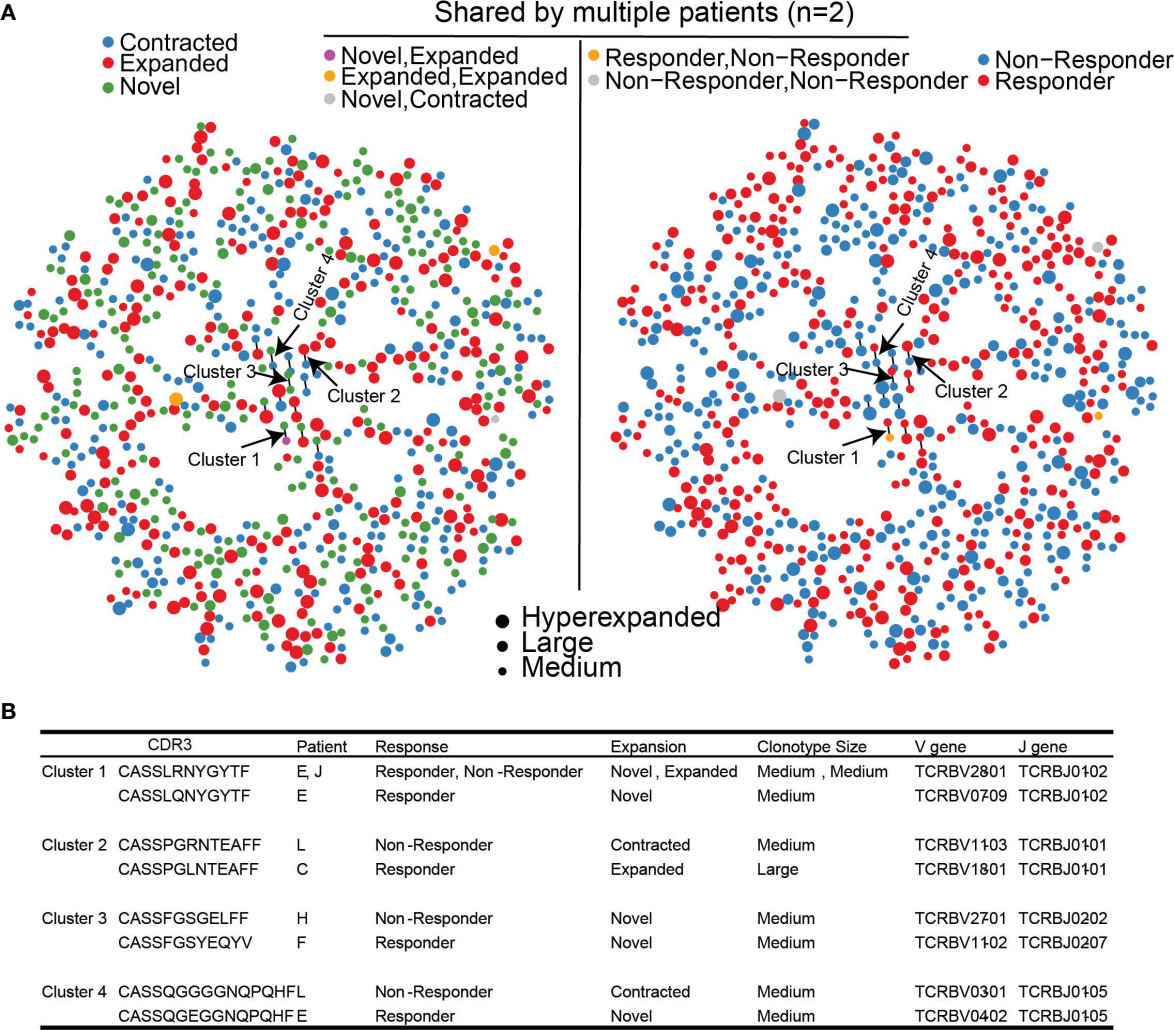
Clustering of significantly changed clonotypes that share similar antigen specificity based on GLIPH2. **(A)** TCR antigen specificity clusters for 780 significantly changed TCR clonotypes. Left panel, dynamic changes of each clonotype after treatment. Right Panel, Clinical outcome of each TCR clonotype. **(B)** Four identified TCR clusters with corresponding clinical outcomes.

Since different clonotypes can recognize the same antigen, we leveraged GLIPH2 which predicts clusters of similar TCR sequences that may recognize the same antigen (24, 25). Among the 168,753 unique clonotypes found in all 11 patients that contained TCRβ chain sequence information, there were 2933/168,753 (1.7%) clonotypes that have been previously reported to recognize viral epitopes based on VDJDB. Of the 939 clonotypes that were significantly changed following treatment, 783 clonotypes (780 unique clonotypes) met the quality assessment based on CDR3 sequence length for GLIPH2 analysis (see methods for details). 20/783 (2.6%) clonotypes were found in the VDJDB recognizing viral antigens suggesting that reported viral clonotypes were largely unchanged with HMA treatment. The majority (756/780; 97%) of significantly changed unique clonotypes harbored unique antigen specificity and only 24/780 (3%) clonotypes formed 12 paired-clusters, suggesting shared antigen specificity (**Figures 5A, B** **)**. The dispersion of clonotype suggests that there was no convergence for the antigen specificity of TCR repertoire. However, among the 12 clusters, there were 4 clusters that were detected in both response groups (**Figure 5B**). None of these clusters contained viral-related clonotypes. Clonotypes of clusters 2 and 4 were contracted in non-responders, but expanded, or emerged, in responders. Interestingly, none of the clonotypes in the 4 clusters were detected in the public TCR database VDJDB (26), which could be attributed to a potentially novel antigen recognition on MDS cells and associated with dynamic changes following HMA treatment, although our analysis cannot confirm this notion.

## Discussion

T-cell dysregulation is implicated in the pathogenesis of MDS (3–5). HMAs, which constitute the mainstay treatment of MDS, have an immunomodulatory role that alter T-cell functions (27, 28). Analysis of the diversity of TCR repertoire can provide valuable information about the underlying T-cell dynamics in response to HMA treatment in MDS.

Compared to healthy bone marrows, MDS patients had less diverse TCR repertoires but higher degree of clonality, suggesting T cell expansion in response to MDS specific antigens. However, unlike previous studies (20, 29, 30), we did not identify significant skewness in CDR3 length of TCRs from MDS patients compared to healthy donors or following HMA treatment. Of note, healthy bone marrow donors were not age-matched to MDS patients and could have impacted the comparison between healthy and MDS bone marrows. Also, previous studies synthesized complimentary DNA from mRNA, compared to our genomic DNA based analysis. The number of mRNA copies per cell is variable, and mRNA is less stable than genomic DNA, which could contribute to the differences (31), although a transcribed TCR may reflect a functional repertoire that is being generated. These factors can lead to bias with respect to the representation of the repertoires as some TCRs can artificially appear more dominant than others (23). Further, antigen-experienced TCR clonotypes may have shorter CDR3 lengths (32). Therefore, our genomic DNA based TCR β chain repertoire profiling can better illustrate the true immune repertoire of MDS. Further, we performed bulk bone marrow analysis rather than on sorted T-cell subsets. Since the TCR repertoire is an aggregate analysis of all T cells, and different T-cell subsets could share same clonotype based on antigen recognition, global analysis provides a more representative characterization of the TCR repertoire in MDS.

While global TCR repertoire analysis did not reveal differences between responders and non-responders to HMA, our individual clonotype analysis revealed an interesting pattern of novel clonotype expansions in responders and clonotype contraction in non-responders, following HMA treatment. The novel clonotypes could represent recruitment of T cells from peripheral tissues or response from bone marrow residing T cells that were below level of sequencing detection prior to treatment. Since pretreatment clonotype profiles were similar in MDS patients, the differential clonotype dynamics is most likely attributed to HMA treatment. However, whether this change is mediated through direct effect of HMAs on T cells or that changes in MDS dynamics in the bone marrow in response to HMA treatment drive T-cell repertoire changes remains to be deciphered. However, our findings provide evidence that responses to HMA treatment in MDS may be mediated *via* T-cell activity.

We also leveraged GLIPH2 analysis that allow identification of clonotype clusters that may recognize the same antigen but have different sequences (24, 25). Our findings revealed that most of the clonotypes are unique, suggesting scarcity of overlap in antigen recognition among clonotypes. However, we identified 4 clonotype clusters with similar antigen recognition profiles but which follow opposite dynamics following treatment between responders and non-responders. These clonotypes were not previously reported in the public database of clonotypes, suggesting that our analysis approach could reveal clonotypes that may play a role in recognizing MDS-specific antigens and requires further experimental validation.

While only 11 patients were analyzed, we used genomic DNA TCR immunosequencing in a homogeneously treated cohort of MDS patients at multiple timepoints following treatment to provide a dynamic profile of the TCR repertoire in MDS. Our findings suggest that T-cell mediated immunity plays a role in response to HMA therapy in MDS patients. Additional studies are required to determine if and how such patients are likely benefit from immunotherapy.

## Data Availability Statement 

The original contributions presented in the study are publicly available. This data can be found here: https://clients.adaptivebiotech.com/pub/abbas-2021-fi.

## Ethics Statement

The studies involving human participants were reviewed and approved by IRB At MD Anderson Cancer Center. The patients/participants provided their written informed consent to participate in this study.

## Author Contributions

HA designed, analyzed, and wrote the study. PR, XJ, and AR analyzed the data with HA. JS, HY, and LL collected bone marrow samples, DNA extraction, and performed sequencing. KC co-supervised analysis of this study. AF and GG-M designed and supervised this study. All authors contributed to the article and approved the submitted version.

## Funding

The work was funded in part by T32 NIH fellowship and Adaptive Biotechnology Young Investigator Award to HA and PR (both were supported by T32 NIH grant).

## Conflict of Interest

The authors declare that the research was conducted in the absence of any commercial or financial relationships that could be construed as a potential conflict of interest.

## Supplementary Material

Supplementary Figure 1 CDR3 length of all patients and by treatment groups.

Supplementary Figure 2 Spectratyping assessment of **(A)** responders and **(B)** non-responders for each timepoint.

Supplementary Figure 3
**(A–K)** Clonotype tracking across the top 10 clonotypes at pre-treatment and their temporal change following treatment.

Supplementary Figure 4
**(A–K)** Clonotype tracking across the top 10 clonotypes at any timepoint and their temporal change during treatment.
