# Supplementary figures and images for: Response to Hypomethylating Agents in Myelodysplastic Syndrome Is Associated With Emergence of Novel TCR Clonotypes
Source: Front Immunol. 2021 Apr 12;12:659625. doi: 10.3389/fimmu.2021.659625 (PMC8072464; doi:10.3389/fimmu.2021.659625)

Supplementary Figure 1t

A.

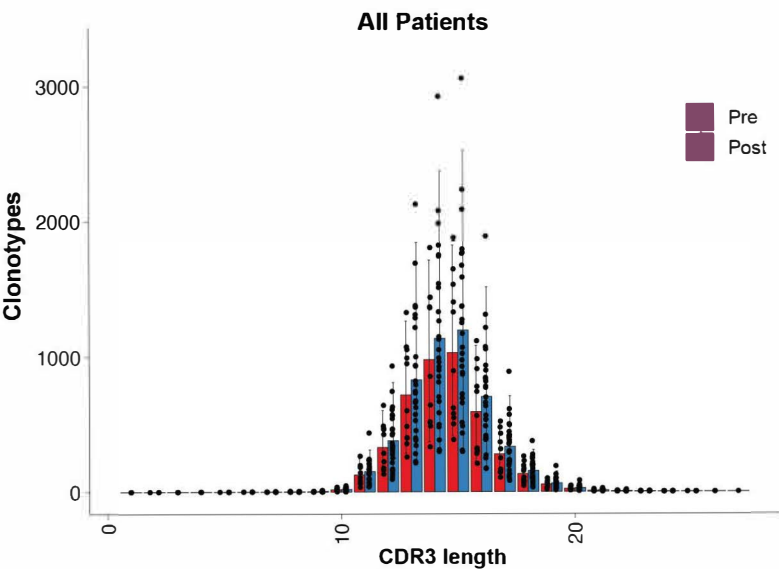

B.

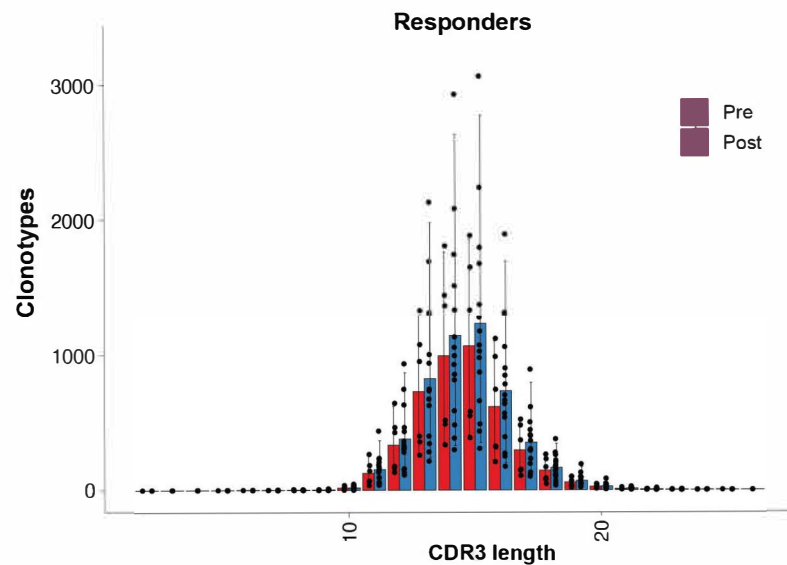

C.

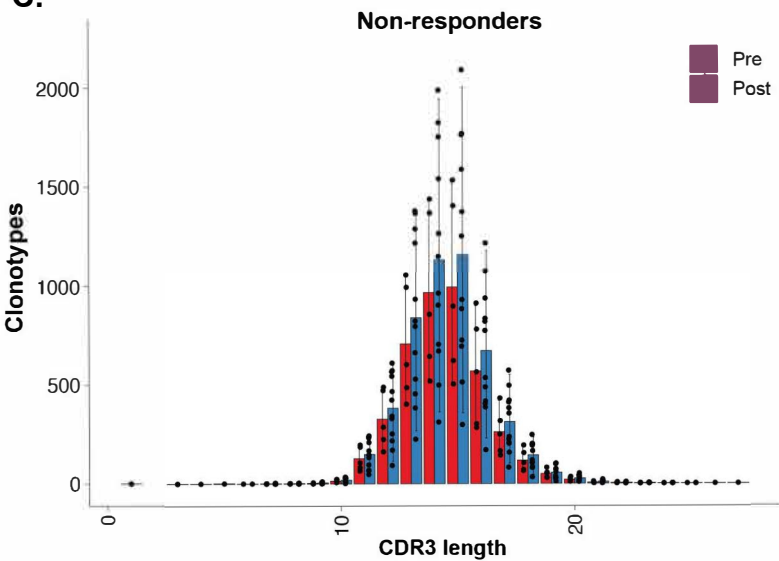

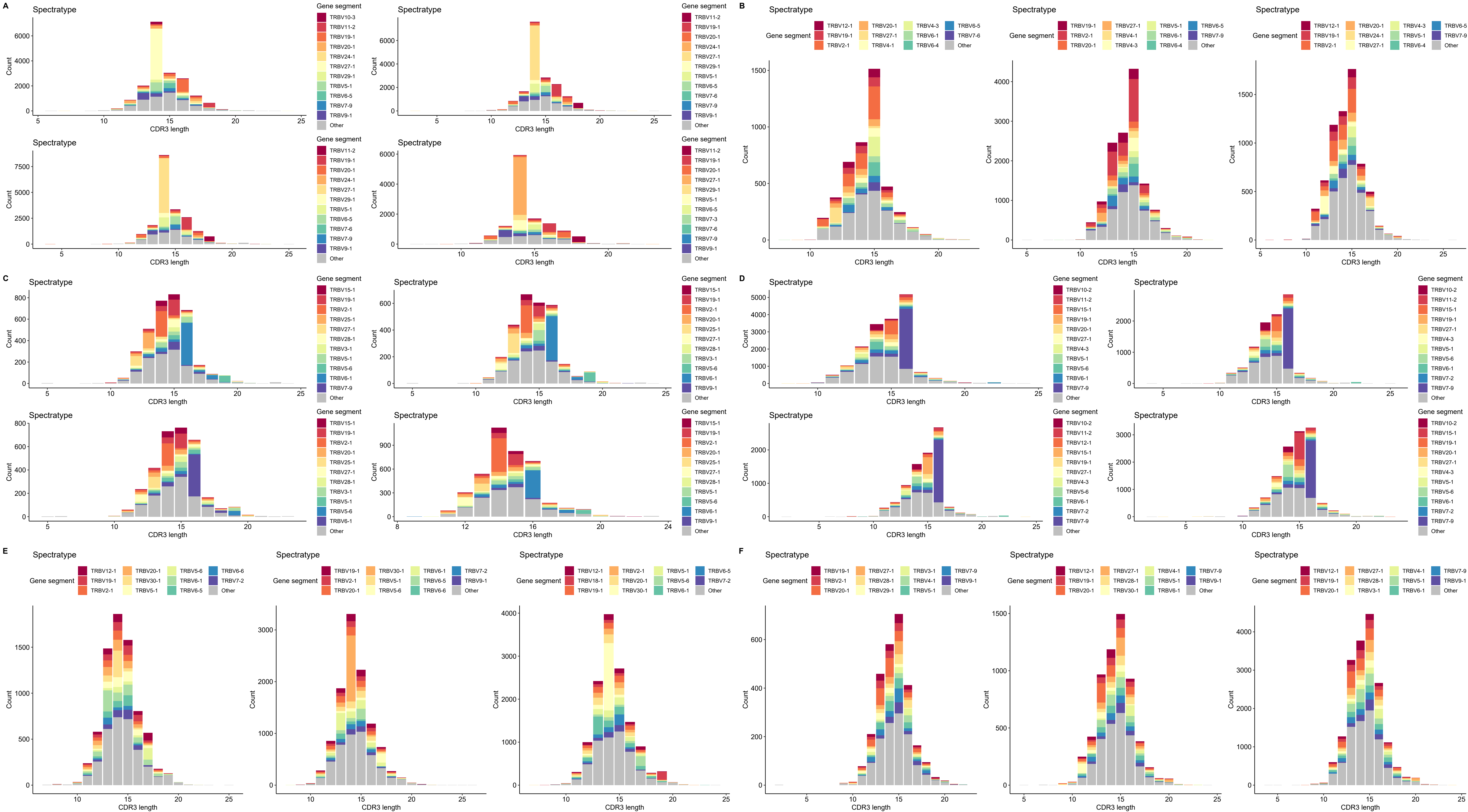

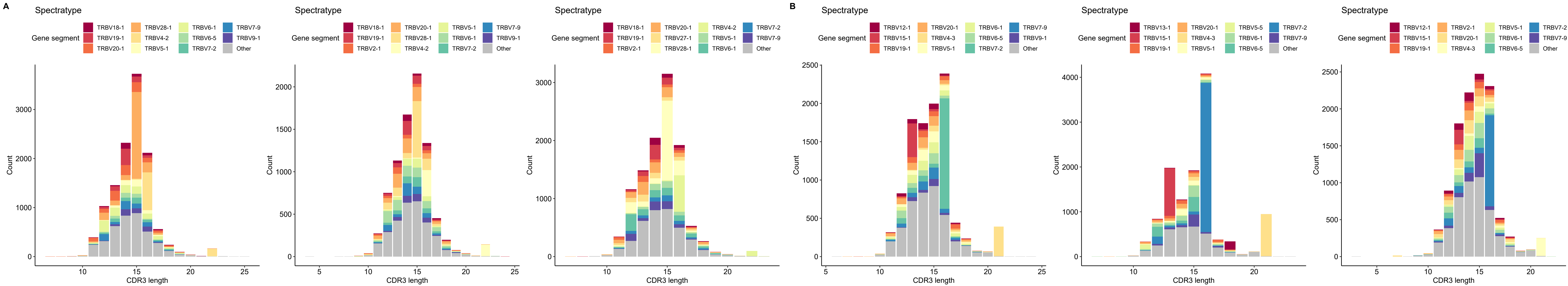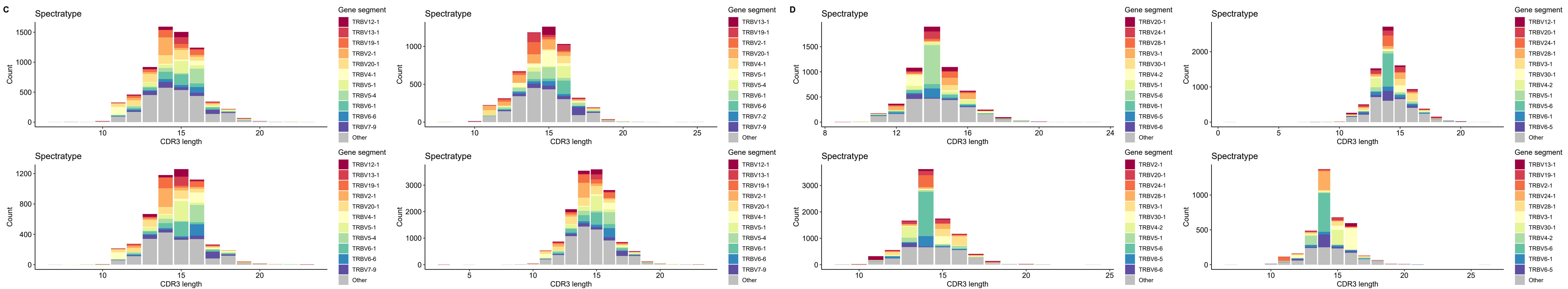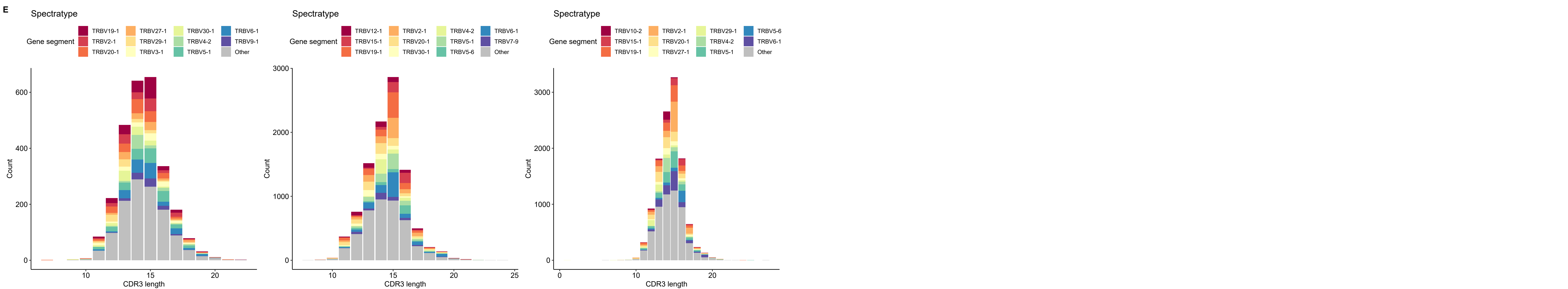

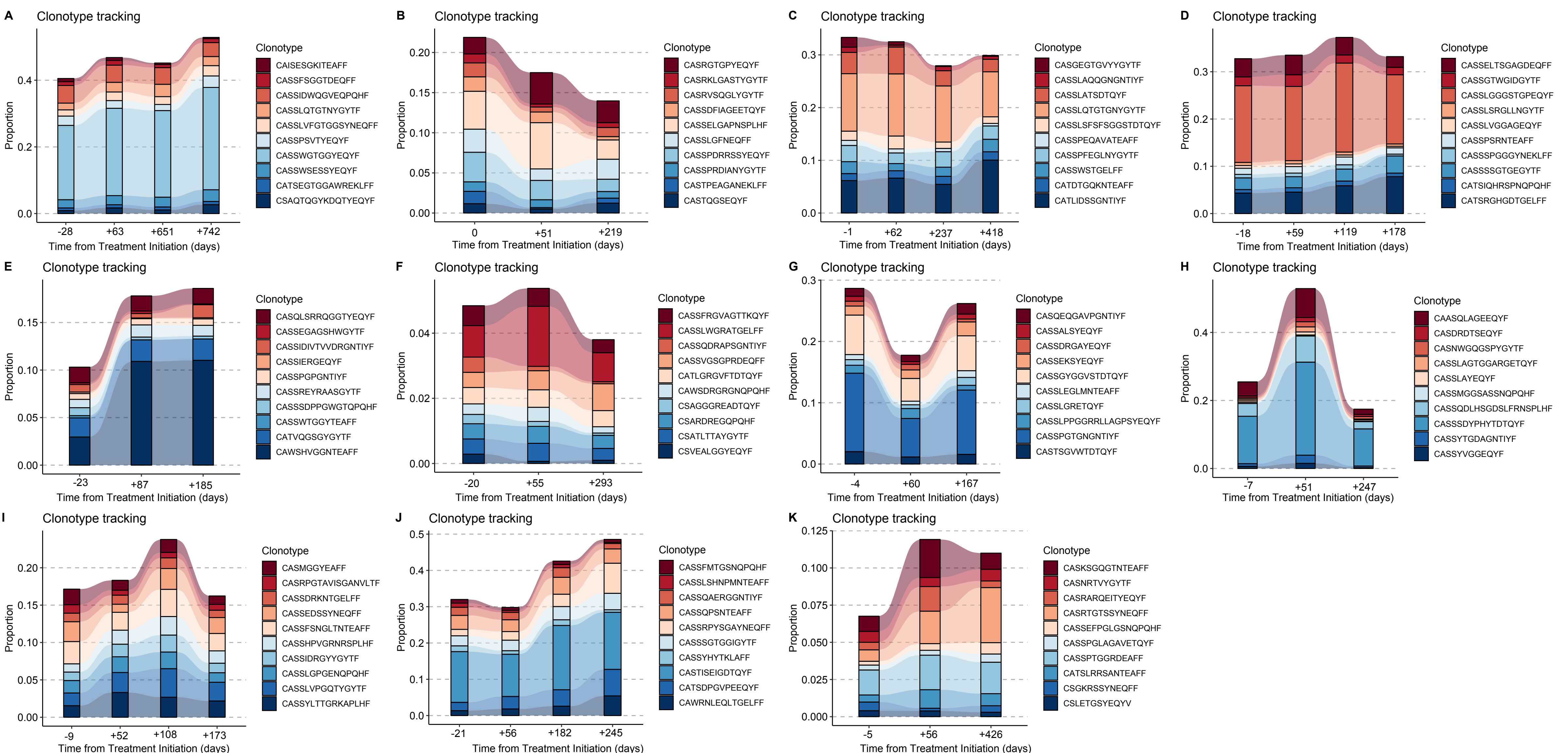

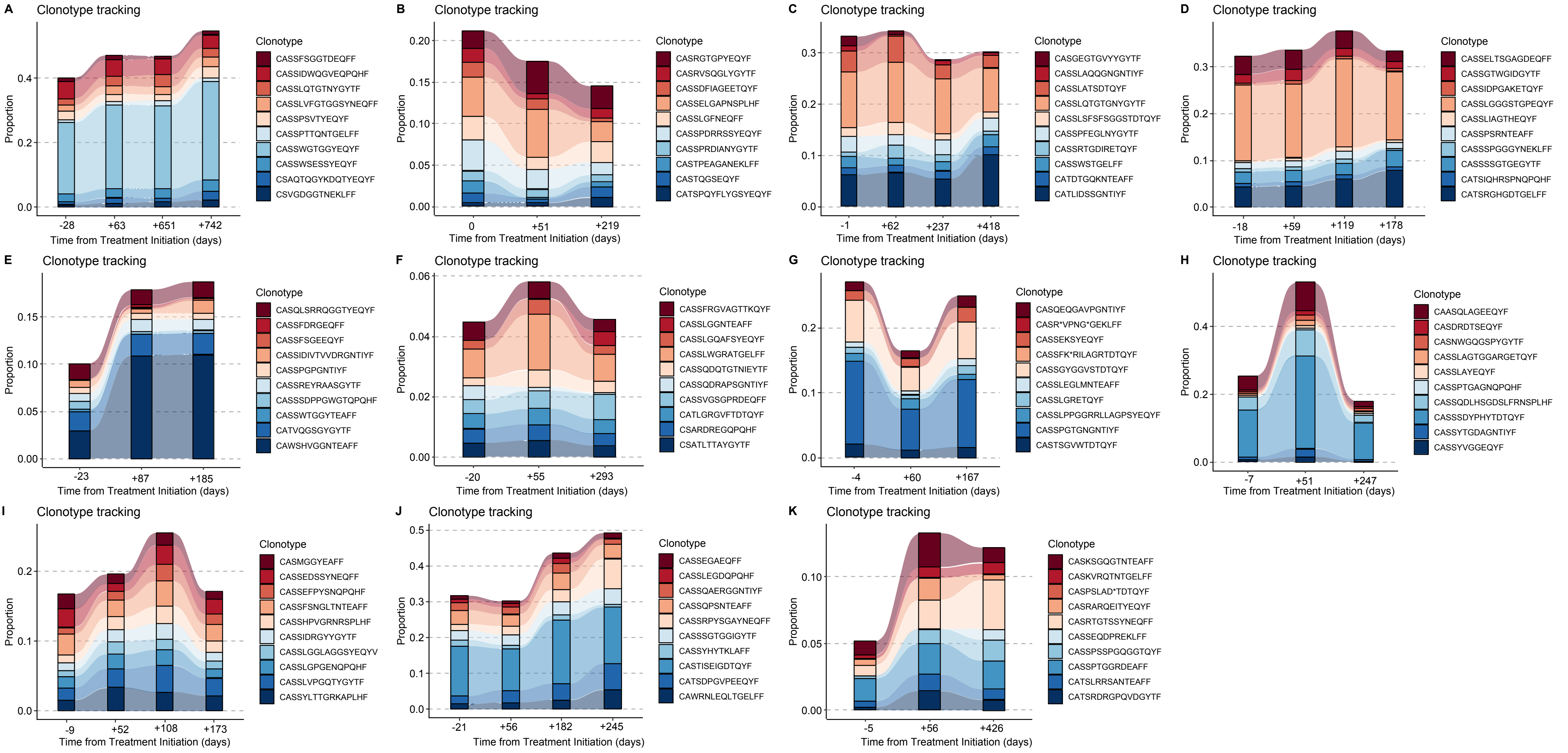

Supplement: Supplementary Figure 1 — CDR3 length of all patients and by treatment groups. [file DataSheet_1.pdf]
